# The application of selenium nanoparticles for enhancing the efficacy of photodynamic inactivation of planktonic communities and the biofilm of *Streptococcus mutans*

**DOI:** 10.1186/s13104-022-05973-w

**Published:** 2022-02-24

**Authors:** Samane Shahmoradi, Aref Shariati, Seyed Mohammad Amini, Nazanin Zargar, Zahra Yadegari, Davood Darban-Sarokhalil

**Affiliations:** 1 Department of Microbiology, School of Medicine, Iran University of Medical Sciences, Hemmat Highway, Next to Milad Tower, Tehran, Iran; 2 Molecular and Medicine Research Center, Khomein University of Medical Sciences, Khomein, Iran; 3 Radiation Biology Research Center, Iran University of Medical Sciences, Tehran, Iran; 4 School of Dentistry, Shahid Beheshti University of Medical Sciences, Tehran, Iran; 5 Department of Dental Biomaterials, Dental School, Shahid Beheshti University of Medical Sciences, Tehran, Iran; 6 Microbial Biotechnology Research Center, Iran University of Medical Sciences, Tehran, Iran

**Keywords:** Selenium nanoparticles, Antimicrobial photodynamic therapy, Combination therapy, Biofilm, *S. mutans*

## Abstract

**Objective:**

*Streptococcus mutans* is one of the principal causative agents of dental caries (tooth decay) found in the oral cavity. Therefore, this study investigates whether selenium nanoparticles (SeNPs) enhance the efficacy of photodynamic therapy (PDT) against both planktonic communities and the one-day-old biofilm of *S. mutans*. In this study, the planktonic and 24-h biofilm of *S. mutans* have been prepared in 96-cell microplates. These forms were treated by methylene blue (MB) and SeNPs and then were exposed to light-emitting diode (LED) lighting. Finally, the results have been reported as CFU/ml.

**Results:**

The outcomes demonstrated that MB-induced PDT and SeNPs significantly reduced the number of planktonic bacteria (*P-*value < 0.001). The comparison between the treated and untreated groups showed that combining therapy with SeNPs and PDT remarkably decreased colony-forming units of one-day-old *S. mutans* biofilm (*P-*value < 0.05). The findings revealed that PDT modified by SeNPs had a high potential to destroy *S. mutans* biofilm. This combination therapy showed promising results to overcome oral infection in dental science.

## Introduction

Dental caries is defined as one of the most tangible chronic oral diseases affecting the health of children and adults [1]. *Streptococcus mutans* are customarily found in various oral cavity sites and are the most common bacterium related to the initiation of dental caries [2, 3]. This bacterium is a Gram-positive coccus, acidogenic, and aciduric bacteria commonly found in the oral cavity. *S. mutans* produces extracellular polysaccharides by fermenting dietary carbohydrates (mainly sucrose). The extracellular polysaccharides have a high adhesion capability to the tooth surface, leading to the oral biofilm. The biofilm formed on tooth surfaces plays a crucial role in dental caries [4, 5].

There are various techniques to control the dental biofilm. However, some of these techniques (e.g., the mechanical disruption of oral biofilms or the use of therapeutic antimicrobials in the oral cavity) are subjected to several restrictions [5]. In this regard, alternative antibacterial strategies (e.g., photodynamic therapy (PDT)) are employed to control microbial growth in the oral cavity [6]. PDT has three principle nontoxic components, including visible light, a photosensitizer (PS), and molecular oxygen. In PDT, light with an appropriate wavelength excited a light-sensitive compound called PS. Then, it induced the formation of reactive oxygen species (ROS) or other singlet oxygen that could kill target cells [7, 8]. However, other particular protective factors like efflux pumps and extracellular polymeric substances (EPS) can affect bacterial biofilm susceptibility toward PDT [9]. Also, the self-aggregation of PS in an aqueous medium may decrease the efficacy of PDT treatment. This issue leads to the lower production of ROS [9, 10]. In this regard, various nanotechnology platforms have been developed to overcome these limitations [11–13].

Recently, several studies reported the antibacterial and anti-biofilm effects of selenium nanoparticles (SeNPs) [14–16]. Also, some studies showed that SeNPs could increase the PDT treatment effect [6, 17]. There is limited data on combine used of SeNPs and PDT; therefore, the present study evaluates the activity of SeNPs in combining with PDT against both planktonic communities and the biofilm of *S. mutans*.

## Main text

### Methods

#### Synthesis and characterization of SeNPs

The chemical reduction method has been utilized to synthesize chitosan-coated SeNPs. The dynamic light scattering (DLS) and transmission electron microscope (TEM) have been employed to characterize the synthesized SeNPs. The synthesis and characterization details of SeNPs have been represented in the previous study performed by the authors. It is essential to note that human fibroblast cells (human gingival fibroblast cells HGF1.PI1 (NCBI C165)) and MTT assay have been used to assess the SeNPs cytotoxicity [17–19].

#### Light source and photosensitizer

In the present study, methylene blue (MB) (Dr. Mojallali, Iran) has been used as the photosensitizing agent. The sterile phosphate-buffered saline (PBS) (pH 7.4) at a concentration of 0.5 mg/ml and membrane filter of 0.22 µm (MS, USA) have been utilized for the solution preparation and filtration procedures, respectively. Finally, the LED lamp (630 nm) (Fotosan 630, Korea, MDD, CMS Dental Denmark) has been employed with an output power of 200 mW/cm^2^ as the light source based on the instruction protocols [20].

#### Assessment of photodynamic deactivation of *S. mutans*

Broth microdilution assays have been used for the analysis process based on the clinical and laboratory standards institute (CLSI) guidelines [21]. Also, the *S. mutans* (PTCC 1683) has been employed as the bacterial standard strains. The wells of 96-well tissue culture plates have been divided into four groups, including LED treatment, MB-induced PDT, PDT with SeNPs, and a control group that did not receive any treatment. In the first step, Brain Heart Infusion (BHI) broth (Merck, Germany) has been used to prepare the fresh bacterial suspension and achieve the turbidity of 0.5 McFarland (1.5 × 10^8^ CFU/ml). Then, the amount of 90 µl of BHI broth was added to all wells. Also, 90 µl of SeNPs and MB were added to the desired wells based on various groups and then were serially diluted. Afterward, the amount of 10 µl of bacteria with a proper concentration (5 × 10^6^ CFU/mL) was added to each well. The microplates were incubated at 37 °C for 15 min, and then the wells were exposed to LED lighting for 1 min. Finally, the amounts of 10 µl of the treated and untreated (control) wells were cultured on BHI agar (Condalab, Spain) and incubated at 37 °C in the presence of 5% CO_2_ for 48 h. After incubation, a colony counter (Teif-Azma Teb, Iran) with an accuracy rate of 10^–4^ has been used for the colony count, showing as CFU/ml. It is necessary to note that in this step, the tests were accomplished in triplicate for each treatment, and the SeNPs concentration with the highest antibacterial effect has been detected and used in the following process.

#### Photodynamic therapy for disrupting the *S. mutans* biofilm

In this section, the *S. mutans* suspension has been prepared for biofilm formation in BHI broth supplemented with 5% sucrose. The amount of 200 µl of bacterial suspensions (0.5 McFarland) was injected into flat-bottomed and sterile 96-well cell culture microplates. Then, they were incubated in a candle jar at 37 °C for 24 h. The bacterial culture medium was carefully aspirated after biofilm formation. Then, all wells were washed twice with a sterile PBS (pH 7.4) to remove bacteria that were not attached to the well. Subsequently, the biofilm treatment has been performed using a single MB (100 µl) or a combination of MB and SeNPs (100 µl), and the plates were incubated at 37 °C for 15 min. Afterward, each well content was removed, and the biofilm was exposed to LED irradiation for 1 min. Then, the amount of 100 µl of fresh BHI broth was added to the wells, and the biofilm was carefully scraped for 30 s, and eventually, the samples were vortexed to be homogenized. Finally, control (untreated) and treated wells were subcultured on the BHI agar and incubated for 48 h with 5% CO_2_ at 37 °C [22, 23].

### Statistical analysis

The PDT and PDTs by SeNPs effects on the planktonic and biofilm forms of *S. mutans* were compared to the control group. In this case, the obtained data were analyzed using ANOVA and Kruskal–Wallis tests. SPSS Statistics version 20.0 (SPSS Inc. Chicago, IL, USA) and GraphPad Prism version 8.3.0 have been employed to implement the statistical analyses. The findings were reported as CFU/ml, and a p-value less than or equal to 0.05 is statistically significant.

## Results

### Photodynamic inactivation of *S. mutans*

The synthesized SeNPs were monodispersed and almost spherical. TEM micrograph was analyzed by digital micrograph. Also, the average size obtained for chitosan-coated SeNPs was 77 ± 27 nm. Besides, the average hydrodynamic diameter of SeNPs based on dynamic light scattering (DLS) measurement and the zeta potential were 80/3 nm and + 70 ± 0.6, respectively. Additionally, the cytotoxicity of SeNPs was evaluated by MTT assay. The authors’ investigation results demonstrated that about 50% of human fibroblast cells were survived at a concentration of 128 µg/ml of SeNPs compared to the untreated group [17].

**Fig. 1:**
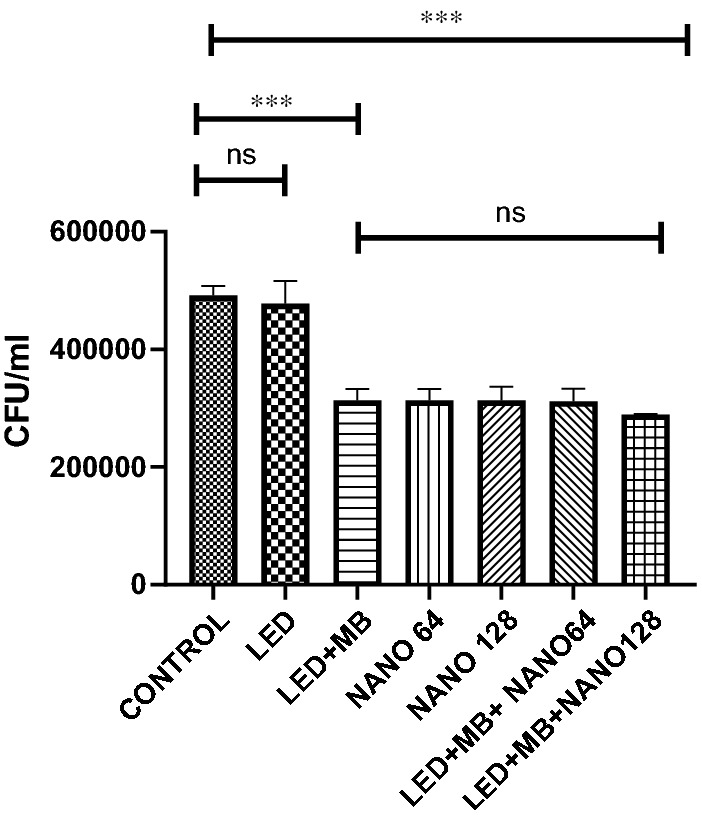
The CFUs of the planktonic form of *S. mutans* after treatment by MB-induced PDT, SeNPs, and combination therapy compared to the control group. (ns: not significant, NANO: SeNPs, MB: methylene blue, *** *P-*value < 0.001)

The outcomes showed that light irradiation alone had no remarkable antibacterial effects against planktonic communities of *S. mutans*. Nevertheless, MB-induced PDT alone and a combination of MB-induced PDT and SeNPs (128 and 64 µg/ml) considerably reduced the number of planktonic *S. mutans* compared to the control group (*p-*value < 0.001). PDT + SeNPs 128 µg/ml showed the highest antibacterial effect. However, a significant difference was not observed between two other groups treated by PDT and combination therapy with PDT + SeNPs 128 µg/ml (Fig. 1).

### Anti-biofilm efficacy of PDT + SeNPs

**Fig. 2:**
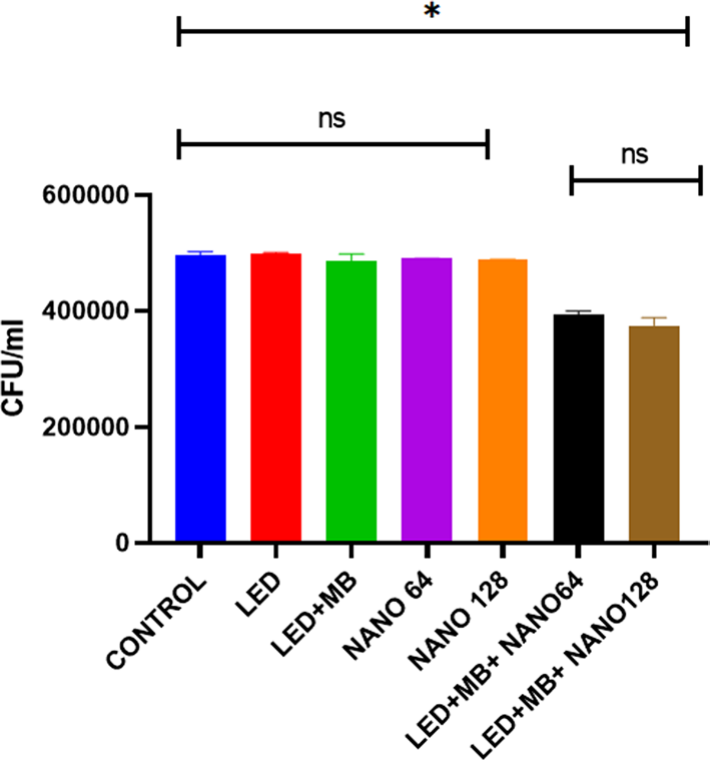
The CFUs of the one-day *S. mutans* biofilm after treatment by MB-induced PDT, SeNPs, and combination therapy compared to the control group. (ns: not significant, NANO: SeNPs, MB: methylene blue, *P-value < 0.05.)

A single MB or a combination of MB and LED had no remarkable effect on 1-day *S. mutans* biofilm (*p-*value > 0.05). Nevertheless, PDT + SeNPs at both concentrations (128 and 64 µg/ml) significantly reduced microbial communities in *S. mutans* biofilm (*p-*value < 0.05). Noteworthy, PDT + SeNPs 128 µg/ml had the highest anti-biofilm effect. But the statistical analysis did not show a considerable difference between this group and other treated groups (Fig. 2).

## Discussion

In this study, the effects of a single PDT and a combination of PDT and SeNPs have been assessed against *S. mutans* planktonic communities and one-day biofilm. Notably, the PDT is known as a host-friendly method used for the inhibition and treatment of oral infections [24, 25]. Since SeNPs have antibacterial and anti-biofilm effects, they can be employed as a powerful option for increasing the efficacy of PDT [26].

The previous studies reported appropriate anti-biofilm activity for MB. It is shown that efflux pumps are very active in bacterial biofilm. Besides, MB is a phenothiazinium salt and acts as a substrate of efflux pumps in bacteria. Therefore, the MB has been employed as a PS in the present study [23, 27, 28]. The LED lamp 630 Fotosan has been selected as the light source. This selection process has been accomplished based on the results of the previous studies. For example, Asnaashari et al. evaluated the antibacterial effect of two types of light sources, including LED lamp 630 nm and a combination of diode laser 810 nm and toluidine blue (TBO) against *Enterococcus faecalis*. Their findings showed that the LED group significantly reduced bacterial load [20]. In another study, the antibacterial activities of LED and helium/neon laser in PDT have been investigated against *E. faecalis* biofilm. The achieved results indicated the same antibacterial effect in both light sources, while some researchers reported that the LED was a more appropriate light source than the complex laser system (due to its simplicity and lower costs of treatment) [29].

The SeNPs with concentrations of 128 and 64 µg/ml had the same antibacterial effect. Indeed, concentration-dependent effects were not observed between SeNPs. Therefore, it is suggested that SeNPs with lower concentrations are used in PDT. It is a proper procedure because of their lower cell cytotoxicity. Also, the findings showed that the combination therapy of PDT and SeNPs remarkably decreased CFUs of *S. mutans* biofilm compared to the use of a single PDT. This result was in agreement with the study conducted by Haris et al. that assessed the effect of the combination of TBO-induced PDT and SeNPs on the *S. mutans* biofilm. Also, their results revealed that SeNPs increased the anti-biofilm effect of PDT [6]. In another study, the effect of TBO-induced combined with silver nanoparticles (TBO-AgNPs**)** has been evaluated on the *S. mutans* biofilm. The results demonstrated that TBO-AgNPs had more phototoxic against *S. mutans* biofilm than the single TBO [13]. Another study assessed the effect of the combination of the MB-mediated antimicrobial photodynamic inactivation (MB-APDI) and chitosan nanoparticles (CSNPs) against *Pseudomonas aeruginosa* and *Staphylococcus aureus* biofilms. The findings indicated that CSNPs boost the activity of MB-APDI. Also, the mixture of MB and CSNPs showed a significant anti-biofilm reduction in the presence of irradiation [12].

Finally, Tran et al. reported that SeNPs showed antibacterial effect against *S. aureus* only after five hours [26]. It is essential to note that Tran et al. studied the effect of SeNPs on planktonic bacteria, while the present study investigates the antibacterial influences of SeNPs on the biofilm of *S. mutans*. The planktonic form of bacteria is more sensitive to medicaments and antimicrobial agents. However, bacteria in biofilm communities had higher resistance to disinfectants than the other group. Therefore, the antibacterial activity of antimicrobial agents on planktonic bacteria is different from that of biofilm [30]. Noteworthy, recent studies that use the combination of PDT and nanoparticles for inhibition of microbial biofilm are listed in Table 1.

**Table 1:** Recent studies that use the combination of photodynamic therapy and nanoparticles for inhibition of microbial biofilm

Year of publication (References)	Nanoparticles	Bacterial species	Outcome
2021 [17]	SeNPs	*Enterococcus faecalis*	Combination therapy remarkably decreased CFUs of one-day-old and root canal biofilm of *E. faecalis* in comparison with the control group
2021 [31]	AgNPs	*Enterococcus faecalis*	Activation with PIPS and PUI increased the AgNPs efficacy irrigating solution for *E. faecalis* elimination from the root canal system
2020 [32]	AgNPs	*Candida species*	PDT with the combination of MB and AgNPs did not have any effect on *C. albicans*. However, this combination therapy decreased the MIC value of *C. parapsilosis*
2020 [33]	AgNPs	*Enterococcus faecalis*	The use of AgNPs leads to an increased in PDT efficacy
2020 [34]	PNP	*Streptococcus mutans*	The combination of PDT with PNP could improve PDT outcomes
2020 [35]	BSA nanoparticles loaded-methylene blue	*Candida albicans*	This drug delivery system could suppress the biofilm formation of *C. albicans*

SeNPs, selenium nanoparticles; CFUs, Colony-Forming Units; AgNPs, Silver nanoparticles; PIPS, passive ultrasonic irrigation; PUI, photon-induced photoacoustic streaming; PDT, Photodynamic therapy; MB, methylene blue; MIC, minimum inhibitory concentration; PNP, Propolis nanoparticle

## Conclusions

The study results revealed that the total number of planktonic bacteria in all treatment groups was remarkably less than in the control group. On the other hand, the *S. mutans* in biofilm communities showed more resistance to the MB-induced PDT. However, SeNPs increased PDT activity and provided a considerable antibiofilm effect against *S. mutans*. Therefore, the results of the present investigation propose that the combination of PDT with SeNPs with the low cytotoxicity effects and the highest antibacterial activities would increase PDT performance, leading to synergistic effects and impairing the biofilm of *S. mutans*. However, more studies are required to determine the exact function of PDT and NPs combination therapy.

## Limitation

The biofilms of *S. mutans* were not formed on dentinal tubules in the present study. So, future studies should evaluate the antibiofilm effect of PDT + SeNPs in the dentine tubule biofilm of *S. mutans*.

## Data Availability

The datasets used and/or analysed during the current study available from the corresponding author on reasonable request.

## Abbreviations

SeNPs: Selenium nanoparticles
PDT: Photodynamic therapy
MB: Methylene blue
LED: Light-emitting diode
PS: Photosensitizer
EPS: Extracellular polymeric substances
DLS: The dynamic light scattering
TEM: Transmission electron microscope

## References

[1] Ren Z, Cui T, Zeng J, Chen L, Zhang W, Xu X, Cheng L, Li M, Li J, Zhou X (2016). Molecule targeting glucosyltransferase inhibits *Streptococcus mutans* biofilm formation and virulence. Antimicrob Agents Chemother.

[2] Lynch DJ, Michalek SM, Zhu M, Drake D, Qian F, Banas JA (2013). Cariogenicity of *Streptococcus mutans* glucan-binding protein deletion mutants. Oral Health Dental Manage.

[3] Alshahrani AM, Gregory RL (2020). In vitro Cariostatic effects of cinnamon water extract on nicotine-induced *Streptococcus mutans* biofilm. BMC Comp Med Therapies.

[4] Klein MI, Hwang G, Santos PH, Campanella OH, Koo H (2015). Streptococcus mutans-derived extracellular matrix in cariogenic oral biofilms. Front Cell Infect Microbiol.

[5] Lin Y, Zhou X, Li Y (2021). Strategies for *Streptococcus mutans* biofilm dispersal through extracellular polymeric substances disruption. Mol Oral Microbiol.

[6] Haris Z, Khan AU (2017). Selenium nanoparticle enhanced photodynamic therapy against biofilm forming *Streptococcus mutans*. Int J Life Sci Sci Res.

[7] Chen P, Yang T, Shi P, Shen J, Feng Q, Su J (2022). Benefits and safety of photodynamic therapy in patients with hilar cholangiocarcinoma: a meta-analysis. Photodiagn Photodynamic Therapy.

[8] Ji B, Wei M, Yang B (2022). Recent advances in nanomedicines for photodynamic therapy (PDT)-driven cancer immunotherapy. Theranostics.

[9] Hung JH, Lee CN, Hsu HW, Ng IS, Wu CJ, Yu CK, Lee NY, Chang Y, Wong TW (2021). Recent advances in photodynamic therapy against fungal keratitis. Pharmaceutics.

[10] Youf R, Müller M, Balasini A, Thétiot F, Müller M, Hascoët A, Jonas U, Schönherr H, Lemercier G, Montier T (2021). Antimicrobial photodynamic therapy: latest developments with a focus on combinatory strategies. Pharmaceutics.

[11] Shrestha A, Hamblin MR, Kishen A (2012). Characterization of a conjugate between Rose Bengal and chitosan for targeted antibiofilm and tissue stabilization effects as a potential treatment of infected dentin. Antimicrob Agents Chemother.

[12] Darabpour E, Kashef N, Mashayekhan S (2016). Chitosan nanoparticles enhance the efficiency of methylene blue-mediated antimicrobial photodynamic inactivation of bacterial biofilms: an in vitro study. Photodiagn Photodyn Ther.

[13] Misba L, Kulshrestha S, Khan AU (2016). Antibiofilm action of a toluidine blue O-silver nanoparticle conjugate on *Streptococcus mutans*: a mechanism of type I photodynamic therapy. Biofouling.

[14] Shoeibi S, Mashreghi M (2017). Biosynthesis of selenium nanoparticles using *Enterococcus faecalis* and evaluation of their antibacterial activities. J Trace Elem Med Biol.

[15] Huang X, Chen X, Chen Q, Yu Q, Sun D, Liu J (2016). Investigation of functional selenium nanoparticles as potent antimicrobial agents against superbugs. Acta Biomater.

[16] Boroumand S, Safari M, Shaabani E, Shirzad M, Faridi-Majidi R (2019). Selenium nanoparticles: synthesis, characterization and study of their cytotoxicity, antioxidant and antibacterial activity. Mater Res Express.

[17] Shahmoradi S, Shariati A, Zargar N, Yadegari Z, Asnaashari M, Amini SM, Darban-Sarokhalil D (2021). Antimicrobial effects of selenium nanoparticles in combination with photodynamic therapy against *Enterococcus faecalis* biofilm. Photodiagn Photodynamic Therapy.

[18] Sundararaju S, Arumugam M, Bhuyar P (2020). *Microbacterium* sp. MRS-1, a potential bacterium for cobalt reduction and synthesis of less/non-toxic cobalt oxide nanoparticles (Co 3 O 4). Beni-Suef Univ J Basic Appl Sci.

[19] Bhuyar P, Rahim MHA, Sundararaju S, Ramaraj R, Maniam GP, Govindan N (2020). Synthesis of silver nanoparticles using marine macroalgae Padina sp. and its antibacterial activity towards pathogenic bacteria. Beni-Suef Univ J Basic Appl Sci.

[20] Asnaashari M, Mojahedi SM, Asadi Z, Azari-Marhabi S, Maleki A (2016). A comparison of the antibacterial activity of the two methods of photodynamic therapy (using diode laser 810 nm and LED lamp 630 nm) against *Enterococcus faecalis* in extracted human anterior teeth. Photodiagn Photodyn Ther.

[21] Wikler MA. Performance standards for antimicrobial susceptibility testing Sixteenth informational supplement. M 100-S 16 2006.

[22] Sharma M, Visai L, Bragheri F, Cristiani I, Gupta PK, Speziale P (2008). Toluidine blue-mediated photodynamic effects on staphylococcal biofilms. Antimicrob Agents Chemother.

[23] Kishen A, Upadya M, Tegos GP, Hamblin MR (2010). Efflux pump inhibitor potentiates antimicrobial photodynamic inactivation of *Enterococcus faecalis* biofilm. Photochem Photobiol.

[24] Garcia MT, Pereira AHC, Figueiredo-Godoi LMA, Jorge AOC, Strixino JF, Junqueira JC (2018). Photodynamic therapy mediated by chlorin-type photosensitizers against *Streptococcus mutans* biofilms. Photodiagn Photodyn Therapy.

[25] Esper MÂLR, Junqueira JC, Uchoa AF, Bresciani E, de Souza Rastelli AN, Navarro RS, de Paiva Gonçalves SE (2019). Photodynamic inactivation of planktonic cultures and *Streptococcus mutans* biofilms for prevention of white spot lesions during orthodontic treatment: an in vitro investigation. Am J Orthod Dentofac Orthop.

[26] Tran PA, Webster TJ (2011). Selenium nanoparticles inhibit *Staphylococcus aureus* growth. Int J Nanomed.

[27] Kvist M, Hancock V, Klemm P (2008). Inactivation of efflux pumps abolishes bacterial biofilm formation. Appl Environ Microbiol.

[28] Zhang L, Mah T-F (2008). Involvement of a novel efflux system in biofilm-specific resistance to antibiotics. J Bacteriol.

[29] Zanin ICJ, Goncalves RB, Junior AB, Hope CK, Pratten J (2005). Susceptibility of *Streptococcus mutans* biofilms to photodynamic therapy: an in vitro study. J Antimicrob Chemother.

[30] Holliday R (2011). Cohen's pathways of the pulp. Br Dent J.

[31] Afkhami F, Ahmadi P, Chiniforush N, Sooratgar A (2021). Effect of different activations of silver nanoparticle irrigants on the elimination of *Enterococcus faecalis*. Clin Oral Invest.

[32] Lavaee F, Yousefi M, Haddadi P (2020). Comparison of the fungicidal efficacy of photodynamic therapy with methylene blue, silver nanoparticle, and their conjugation on oral Candida isolates using cell viability assay. Curr Med Mycol.

[33] Aydın H, Er K, Kuştarcı A, Akarsu M, Gençer GM, Er H, Felek R (2020). Antibacterial activity of silver nanoparticles activated by photodynamic therapy in infected root canals. Dental Med Problems.

[34] Afrasiabi S, Pourhajibagher M, Chiniforush N, Bahador A (2020). Propolis nanoparticle enhances the potency of antimicrobial photodynamic therapy against *Streptococcus mutans* in a synergistic manner. Sci Rep.

[35] Ambrósio JAR, Pinto B, da Silva BGM, Passos J, Beltrame Junior M, Costa MS, Simioni AR (2020). BSA nanoparticles loaded-methylene blue for photodynamic antimicrobial chemotherapy (PACT): effect on both growth and biofilm formation by *Candida albicans*. J Biomater Sci Polym Ed.

